# Association between tobacco smoking and prevalence of HIV, tuberculosis, hypertension and diabetes in rural South Africa: a cross-sectional study

**DOI:** 10.1186/s12889-024-20791-4

**Published:** 2024-11-27

**Authors:** Glory Chidumwa, Stephen Olivier, Hloniphile Ngubane, Thando Zulu, Mareca Sithole, Resign Gunda, The Vukuzazi Team, Ronel Sewpaul, Gina Kruse, Nancy A. Rigotti, Willem A. Hanekom, Mark J. Siedner, Krishna P. Reddy, Emily B. Wong

**Affiliations:** 1 Africa Health Research Institute, Durban & Somkhele, KwaZulu-Natal South Africa; 2 Wits RHI, University of the Witwatersrand, Johannesburg, South Africa; 3 Human & Social Capabilities Division, Human Sciences Research Council, Cape Town, South Africa; 4 Massachusetts General Hospital, Boston, MA USA; 5 Harvard Medical School, Boston, MA USA; 6 Division of Infection and Immunity, University College London, London, UK; 7 Division of Infectious Diseases, Heersink School of Medicine, University of Alabama at Birmingham, Birmingham, AL USA

**Keywords:** Tobacco, Smoking, Communicable diseases, Non-communicable diseases

## Abstract

**Background:**

South Africa is facing a convergence of communicable diseases (CDs) and non-communicable diseases (NCDs). There are limited data about how tobacco use contributes to the burden of these conditions, especially in rural populations.

**Methods:**

We analyzed the associations between current tobacco smoking and four important CDs and NCDs in Vukuzazi, a cross-sectional study of individuals aged 15 years and older conducted between 2018–2020 in a demographic surveillance area in KwaZulu-Natal, South Africa. Data on HIV, active tuberculosis (TB), hypertension and diabetes mellitus were collected via direct measurement from participants.

**Results:**

Of 18,024 participants (68% female, median age 37 years [interquartile range 23–56 years]), 1,301 (7.2%) reported current smoking. Prevalence of HIV infection was similarly high among people who currently smoked (34.6%) and people who had never smoked (33.9%). However, among people living with HIV (PLWH), there was a higher prevalence of detectable viremia in people reporting current smoking compared to people who reported never smoking (28.8% vs. 16.6%; *p*-value < 0.001). Active TB was more prevalent in people who currently smoked than in people who never smoked (3.1% vs 1.3%, *p* < 0.001). In contrast, the prevalence of hypertension and diabetes mellitus were lower in people reporting current smoking than in people reporting never smoking (17.1% vs 26.0% (*p* < 0.001), and 2.5% vs 10.2% (*p* < 0.001), respectively). In sex-stratified multiple logistic regression analyses that were adjusted for potential confounding factors (including body mass index for the NCDs), the magnitude of differences in CD prevalence between people who currently smoked and people who never smoked decreased, whereas the lower prevalence of NCDs among people reporting current smoking persisted.

**Conclusions:**

In rural South Africa, smoking is associated with higher prevalence of active TB, and people with HIV who smoke have worse disease control. In contrast, hypertension and diabetes mellitus are less common in those who smoke. Interventions to screen for TB among those who smoke and to address smoking among people with HIV may be particularly impactful.

**Supplementary Information:**

The online version contains supplementary material available at 10.1186/s12889-024-20791-4.

## Introduction

South Africa faces the challenge of co-occurring epidemics of communicable disease (CDs) and non-communicable diseases (NCDs) [1]. We have previously reported on the Vukuzazi study, conducted between May 2018 and March 2020, which estimated the prevalence and overlap of four common and treatable CDs and NCDs (HIV, tuberculosis [TB], hypertension and diabetes mellitus) among individuals aged 15 years and older in a population in KwaZulu-Natal, South Africa [2]. In this cohort, at least one of these four conditions was present in 52.1% of the > 18,000 participants, and 11.8% had two or more active conditions.

Tobacco use is the leading preventable cause of death globally and disproportionately impacts the health of people in low and middle-income countries (LMICs), where 80% of people who use tobacco live [3]. In the United States of America, among people living with HIV (PLWH) on antiretroviral therapy, smoking is now expected to reduce life expectancy more than HIV itself [4]. Similarly, tobacco smoking has been associated with poor TB outcomes, including higher mortality [5, 6]. Tobacco smoking, like hypertension and diabetes mellitus, is a major risk factor for cardiovascular disease and death [7–9].

In South Africa, due to public health efforts to increase access to prevention and treatment, morbidity and mortality due to HIV and TB have been on the decline, while NCDs morbidity and mortality are rising nationally [10–12]. On the other hand, there is evidence suggesting that smoking prevalence is increasing in South Africa nationally (https://southafrica.tobaccocontroldata.org/en/home/prevalence/), though high prevalence has been reported among men [13]. In addition, evidence has shown that the prevalence of active smoking is high among patients with hypertension or diabetes in Africa, with the heaviest burden in Northern Africa [14]. However, there are limited data about the intersection of tobacco use with chronic diseases in sub-Saharan Africa, where CDs, such as HIV and TB, and NCDs, such as hypertension and diabetes mellitus, are widely prevalent. We sought to investigate the association between tobacco smoking with CDs and NCDs in rural South Africa using data from the Vukuzazi cohort.

## Methods

### Study participants and setting

The Vukuzazi population-wide health screening study was conducted among people 15 years of age and older by the Africa Health Research Institute in uMkhanyakude district in northern KwaZulu-Natal, South Africa between 2018–2020. Most of the uMkhanyakude area is poor and rural, with several informal peri-urban settlements and a single urban township [15]. Details of the Vukuzazi study were published previously [16]. Vukuzazi is nested within the AHRI’s Population Intervention Programme (PIP) study, which allows for both demographic and health system follow-up of participants [17]. Participants are observed prospectively three times per year through the PIP demographic surveillance activities, which records changes to their household residence, demographic and vital status to understand population and health dynamics of a rural community [17]. In brief, study measurements included HIV testing with an immunoassay and HIV-1 RNA viral load (in people with a positive immunoassay). TB screening was conducted using symptom screen and digital chest x-ray, and for people with abnormalities on either of these, sputum was collected for molecular (GeneXpert MTB/RIF) and microbiological (MGiT) testing [18]. Research nurses took three blood pressure readings, with 5 min resting time in between, after 30 min of inactivity, using portable electronic devices (OMRON Healthcare, Model M6), following the World Health Organization STEPwise protocol as described previously [16]. Hemoglobin A1c was measured by collecting whole blood into ethylenediaminetetraacetic acid tubes. Blood pressure and hemoglobin A1c were measured as indicators of hypertension and diabetes mellitus, respectively. All measurements were conducted at a single study visit at the household level. The questionnaire used in this study has previously been published elsewhere [16].

### Exposure variable

Our primary exposure of interest was self-reported tobacco smoking, categorized as current (answering “Yes” to the question “Do you currently smoke any tobacco products, such as cigarettes, cigars or pipes?”), former (ever smoked tobacco and quit smoking at least one year prior to survey) or never (never smoked any tobacco products in the past). In the current analysis, we were interested in combustible tobacco given previously described associations with tuberculosis and cardiovascular diseases in other settings [5, 19].

### Outcome variables

Our primary outcomes of interest were the presence of the following conditions: (1) HIV, defined as participants with a positive HIV immunoassay; (2) active TB, defined as on TB treatment or with newly diagnosed sputum-positive TB (GeneXpert MTB/RIF and/or MGiT culture positive); (3) hypertension, defined as self-reported disease (people who reported having received the diagnosis from a health professional and were on active medication for the condition) and/or by measurements of blood pressure (BP) (systolic BP ≥ 140 mm Hg and/or diastolic BP ≥ 90 mm Hg); and (4) diabetes mellitus, defined as self-reported disease (people who reported having received the diagnosis from a health professional and were on active medication for the condition) and/or hemoglobin A1c > 6.5% [16]. Among PLWH, we further defined whether individuals had detectable HIV viremia (≥ 400 copies/mL) or undetectable HIV viral load (< 400 copies/mL) [20].

### Covariates

We included the following variables as covariates to account for potential confounding between smoking and disease prevalence: age, measured body mass index (BMI, categorized as < 18.5 kg/m^2^: underweight, 19–24.9 kg/m^2^: normal, 25–29.9 kg/m^2^: overweight, ≥ 30 kg/m^2^: obese), personal economic activity (employed, unemployed, or part-time), and household socio-economic status (SES, estimated through a principal components analysis of asset ownership) [21].

### Statistical analysis

This analysis excluded Vukuzazi participants who did not answer the smoking status questions. To assess the relationship between smoking and CDs and NCDs, we calculated and compared the percent of participants with each disease, stratified by smoking status (current, former and never). Next, we utilized multiple logistic regression to compare current smoking with never smoking for each of the outcomes. Alternative analyses were conducted comparing (a) ever smoking (defined as current and former smoking) and never smoking and (b) current smoking with not current smoking (defined as never and former smoking). Based on published literature, we determined, a priori, candidate variables including age, BMI, household socio-economic status to be included in the models [2]. Multiple logistic regression models were stratified by sex because of the extreme over-representation of males in the current smoking group. We used Stata Special Edition (version 17.1) program.

## Results

Of the 18,024 participants in the Vukuzazi cohort (68% female, median age 37 years [interquartile range 23–56 years]), 1,301 (7.2%) reported current smoking, 150 (0.8%) reported former smoking and 16,573 (92%) reported never smoking. Of those who currently smoked, 1069 (82%) reported smoking cigarettes, 175 (13%) smoking cigars, 23 (1.8%) smoking pipes, 441 (33.9%) smoking bidis, and 37 (2.8%) smoking hukkah either daily or weekly. Table 1 shows the demographic characteristics of the participants by smoking status. Among people who reported current smoking, the median age was 37 years (interquartile range 27–49 years) and the majority were male (*n* = 1,177; 90.5%). Compared to people reporting former or never smoking, people reporting current smoking were more likely to be male, younger, and less overweight or obese. People who reported former smoking were older, more likely to be unemployed, and more likely to be in the poorest household socio-economic status quintile.

**Table 1:** Participant characteristics, by smoking category (*n* = 18,024)

**Characteristic (n, %)**	**Total**	**Current smoking**	**Former smoking**	**Never smoking**	***P*-value**
	(*N* = 18,024)	(*N* = 1,301)	(*N* = 150)	(*N* = 16,573)	
**Sex**					< 0.001
Male	5800 (32.2%)	1,177 (90.5%)	101 (67.3%)	4,522 (27.3%)	
Female	12224 (67.8%)	124 (9.5%)	49 (32.7%)	12,051 (72.7%)	
**Age category at enrolment, years**					< 0.001
15–44	10955 (60.8%)	872 (67.0%)	58 (38.7%)	10,025 (60.5%)	
> 45	7069 (39.2%)	429 (33.0%)	92 (61.3%)	6,548 (39.5%)	
**BMI category**^**a**^					< 0.001
Underweight	857 (4.8%)	136 (10.6%)	10 (6.8%)	711 (4.3%)	
Normal	7053 (39.5%)	879 (68.4%)	69 (47.3%)	6,105 (37.2%)	
Overweight	4048 (22.7%)	179 (13.9%)	33 (22.6%)	3,836 (23.4%)	
Obese	5884 (33.0%)	92 (7.2%)	34 (23.3%)	5,758 (35.1%)	
**Personal economic activity**					< 0.001
Employed	2758 (18.0%)	281 (22.6%)	20 (14.8%)	2,457 (17.6%)	
Part-time	617 (4.0%)	79 (6.4%)	7 (5.2%)	531 (3.8%)	
Not employed	11965 (78.0%)	884 (71.1%)	108 (80.0%)	10,973 (78.6%)	
**Household socio-economic status quintile**					0.031
1 (poorest)	2693 (15.2%)	237 (18.5%)	30 (20.4%)	2,417 (14.9%)	
2	3606 (20.4%)	268 (20.9%)	28 (19.0%)	3,298 (20.3%)	
3	3839 (21.7%)	264 (20.6%)	30 (20.4%)	3,559 (21.9%)	
4	3951 (22.3%)	269 (21.0%)	31 (21.1%)	3,638 (22.4%)	
5 (wealthiest)	3600 (20.4%)	244 (19.0%)	28 (19.0%)	3,341 (20.6%)	

All values are frequencies are reported with percentages in parenthesis

*P*-values shown are from Chi-Square tests for smoking category (including former smoking) with demographic characteristics

^a^BMI: Less than 18.5 = Underweight, 18.5 to 24.9 = Healthy, 25 to 29.9 = Overweight and 30 or higher = Obese

The percentage of participants with HIV was similarly high among the three smoking categories (current 34.6%, former 33.6%, never 33.9%; *p*-value = 0.879) (Table 2). However, among PLWH, detectable viremia was more common in people reporting current smoking compared to people reporting former or never smoking (28.8% vs. 12.0% vs. 16.6%; *p*-value < 0.001). The percentage of participants with active TB was significantly higher among people reporting current or former smoking (current, 3.1%; former, 4.7%) compared to people who reported never having smoked (1.3%, *p*-value < 0.001). In contrast, hypertension and diabetes mellitus were less common in people reporting current smoking (17.1% and 2.5%, respectively) than people reporting former smoking (31.5% and 14.8%) or never smoking (26.2% and 10.2%, *p*-values for both hypertension and diabetes mellitus were < 0.001). Stratification of these data by sex revealed that among males, the percentage of participants who had each of the conditions defined (HIV, HIV with detectable viremia, active TB, hypertension and diabetes mellitus) all differed significantly by smoking status (Table 3). This includes a significantly higher prevalence of HIV in males reporting current smoking compared with males reporting former or never smoking. In contrast, in females, there were no significant differences in CDs or NCDs by smoking status (Supplementary Table 1).

**Table 2:** Communicable and non-communicable diseases by smoking category, both sexes

**Variable**	**Total**	**Current smoking**	**Former smoking**	**Never smoking**	
	(*N* = 18,024)	(*N* = 1,301)	(*N* = 150)	(*N* = 16,573)	***P*-value**
**HIV ELISA result**					0.879
Positive	6,096 (34.0%)	448 (34.6%)	50 (33.6%)	5,598 (33.9%)	
Negative	11,853 (66.0%)	847 (65.4%)	99 (66.4%)	10,907 (66.1%)	
**Viral load, among those with HIV**					< 0.001
< 400 copies/mL	5,019 (82.4%)	318 (71.0%)	44 (88.0%)	4,657 (83.2%)	
>= 400 copies/mL	1,074 (17.6%)	130 (29.0%)	6 (12.0%)	938 (16.8%)	
**Active tuberculosis**					< 0.001
Yes	245 (1.5%)	38 (3.1%)	6 (4.7%)	201 (1.3%)	
No	16,549 (98.5%)	1,179 (96.9%)	123 (95.3%)	15,237 (98.7%)	
**Hypertension**					< 0.001
Yes	4,603 (25.6%)	222 (17.1%)	47 (31.5%)	4,334 (26.2%)	
No	13,373 (74.4%)	1,076 (82.9%)	102 (68.5%)	12,195 (73.8%)	
**Diabetes mellitus**					< 0.001
Yes	1,737 (9.7%)	32 (2.5%)	22 (14.8%)	1,683 (10.2%)	
No	16,216 (90.3%)	1,262 (97.5%)	127 (85.2%)	14,827 (89.8%)	

*P*-values shown are from Chi-Square tests for smoking category (including former smoking) with communicable diseases and non-communicable diseases. All values are frequencies reported with percentages in parentheses. There were no significant differences between those who did and did not have responses for the TB and hypertension outcomes

**Table 3:** Communicable and non-communicable diseases by smoking category for males

**Variable**	**Total**	**Current smoker**	**Former smoking**	**Never smoker**	
	(*N* = 5,800)	(*N* = 1,177)	(*N* = 101)	(*N* = 4,522)	***P*-value**
**HIV ELISA result**					< 0.001
Positive	1,406 (24.4%)	391 (33.4%)	28 (27.7%)	987 (21.9%)	
Negative	4,365 (75.6%)	780 (66.6%)	73 (72.3%)	3,512 (78.1%)	
**Viral load category**					0.012
< 400 copies/mL	1,051 (73.7%)	275 (69.8%)	26 (92.9%)	750 (74.7%)	
> = 400 copies/mL	375 (26.3%)	119 (30.2%)	2 (7.1%)	254 (25.3%)	
**Active tuberculosis**					0.001
Yes	111 (2.1%)	35 (3.2%)	5 (5.5%)	71 (1.7%)	
No	5,246 (97.9%)	1,067 (96.8%)	86 (94.5%)	4,085 (98.3%)	
**Hypertension**					0.001
Yes	1,042 (18.0%)	189 (16.1%)	31 (31.0%)	822 (18.2%)	
No	4,743 (82.0%)	986 (83.9%)	69 (69.0%)	3,688 (81.8%)	
**Diabetes mellitus**					< 0.001
Yes	315 (5.5%)	24 (2.1%)	13 (12.9%)	278 (6.2%)	
No	5,455 (94.5%)	1,146 (97.9%)	88 (87.1%)	4,221 (93.8%)	

*P*-values shown are from Chi-Square tests for smoking category (including former smoking) with communicable diseases and non-communicable diseases. All values are frequencies reported with percentages in parentheses

Multiple logistic regression models comparing percentage of participants with CDs and NCDs among people who ever smoked and never smoking status, stratified by sex and adjusted for potential confounders (age and socio-economic status), are shown in Table 4. Former smokers were included with current smokers in the multiple logistic regression models since only 150 participants (0.8%) were former smokers. In the multiple logistic model, we found no relationship between being HIV-positive and ever smoking in either males or females (adjusted odds ratio [aOR] = 1.09, 95% confidence interval [CI]: 0.93–1.30, *p* = 0.295; and aOR = 1.32, 95% CI: 0.93–1.87, *p* = 0.118, respectively), nor did we find any relationship between viremic HIV and ever smoking in either males or females (aOR = 0.89, 95% CI 0.66–1.19, *p* = 0.424; and aOR = 0.91, 95% CI 0.50–168, *p* = 0.774, respectively). In males, we found an increased odds of active TB among people reporting ever smoking compared with never smoking (aOR = 2.07, 95% CI: 1.32–3.23, *p* = 0.001). Also in males, ever smoking was associated with lower odds of hypertension (aOR = 0.68,,95% CI: 0.56–0.84, *p*-value < 0.001) and diabetes mellitus (aOR = 0.54, 95% CI: 0.36–0.79, *p*-value = 0.002) compared with never smoking. In females, there was no increase in odds of active TB, hypertension or diabetes mellitus in people reporting ever smoking compared with people reporting never smoking. To better understand the relationship between body weight, tobacco use and chronic disease, we included BMI in the multiple logistic regression (Table 5). The direction of all relationships held true in this analysis. We further found similar relationships when comparing current smokers with never smokers (Supplementary Table 2 and Supplementary Table 3, including BMI in the model), excluding former smokers. In addition, we found similar results from an alternative analysis performed to group former smoking with never smokers in Supplementary Table 4 (current smoking versus ever smoking) and Supplementary Table 5 (current smoking versus not current smoking, including BMI in the model).

**Table 4:** Association between communicable and non-communicable diseases and ever smoking, multiple logistic regression (former smokers included with current smokers)

Variable	Males		Females	
	**Adjusted odds ratio (95% CI)**	***P*-value**	**Adjusted odds ratio (95% CI)**	***P*-value**
**HIV (positive ELISA result)**	**Prevalence = 1,372/5,661 (24.2%)**		**Prevalence = 4,582/11,955 (38.3%)**	
Never smoking	Reference		Reference	
Ever smoking	1.09 (0.93; 1.30)	0.295	1.32 (0.93; 1.87)	0.118
**Viral load < 400 copies/mL among those with HIV**	**Prevalence = 1,027/1,392 (73.8%)**		**Prevalence = 3,922/4,610 (85.1%)**	
Never smoking	Reference		Reference	
Ever smoking	0.89 (0.66; 1.19)	0.424	0.91 (0.50; 1.68)	0.774
**Active tuberculosis**	**Prevalence = 110/5,690 (1.9%)**		**Prevalence = 131/11,891 (1.1%)**	
Never smoking	Reference		Reference	
Ever smoking	2.07 (1.32; 3.23)	0.001	1.97 (0.69; 5.65)	0.205
**Hypertension**	**Prevalence = 1,017/5,675 (17.2%)**		**Prevalence = 3,504/11,967 (29.3%)**	
Never smoking	Reference		Reference	
Ever smoking	0.68 (0.56; 0.84)	< 0.001	0.94 (0.62; 1.41)	0.755
**Diabetes mellitus**	**Prevalence = 307/5,660 (5.4%)**		**Prevalence = 1,400/11,960 (11.7%)**	
Never smoking	Reference		Reference	
Ever smoking	0.54 (0.36; 0.79)	0.002	1.02 (0.59; 1.76)	0.936

For each outcome variable (HIV, viral load < 400 copies/mL, tuberculosis, hypertension, and diabetes mellitus), we ran separate multiple logistic regression models, adjusting for age and household socio-economic status

*CI* Confidence Interval

**Table 5:** Association between communicable and non-communicable diseases and ever smoking, multiple logistic regression, including BMI (former smokers included with current smokers)

Variable	Males		Females	
	**Adjusted odds ratio (95% CI)**	***P*-value**	**Adjusted odds ratio (95% CI)**	***P*-value**
**HIV (positive ELISA result)**	**Prevalence = 1,361/5,601 (24.3%)**		**Prevalence = 4,566/11,837 (38.6%)**	
Never smoking	Reference		Reference	
Ever smoking	0.99 (0.83; 1.17)	0.883	1.16 (0.82; 1.66)	0.398
**Viral load < 400 copies/mL among those with HIV**	**Prevalence = 1,016/1,381 (73.6%)**		**Prevalence = 3,906/4,593 (85.0%)**	
Never smoking	Reference		Reference	
Ever smoking	0.92 (0.68; 1.25)	0.612	0.91 (0.49; 1.18)	0.762
**Active tuberculosis**	**Prevalence = 108/5,629 (1.9%)**		**Prevalence = 131/11,773(1.1%)**	
Never smoking	Reference		Reference	
Ever smoking	1.78 (1.12; 2.81)	0.014	1.62 (0.56; 4.64)	0.370
**Hypertension**	**Prevalence = 989/5,614 (17.6%)**		**Prevalence = 3,422/11, 848 (28.9%)**	
Never smoking	Reference		Reference	
Ever smoking	0.77 (0.62; 0.95)	0.016	1.09 (0.72; 1.66)	0.679
**Diabetes mellitus**	**Prevalence = 298/5,600 (5.3%)**		**Prevalence = 1,366/11,842 (11.5%)**	
Never smoking	Reference		Reference	
Ever smoking	0.69 (0.46; 1.04)	0.077	1.34 (0.77; 2.34)	0.301

For each outcome variable (HIV, viral load < 400 copies/mL, tuberculosis, hypertension, and diabetes mellitus), we ran separate multivariable logistic regression models, adjusting for age, body mass index, and household socio-economic status

*CI* Confidence Interval

## Discussion

In a large population-based cohort in rural South Africa, viremic HIV and active TB were more common, and hypertension and diabetes mellitus were less common, in people reporting current smoking compared to never smoking. In this setting, over 90% of people reporting current smoking were males. Multiple logistic regression analyses, adjusted for potential confounding factors, indicated that among males, current smoking was associated with increased odds of active TB and decreased odds of hypertension and diabetes mellitus compared to never smoking.

The high prevalence of HIV in this population highlights the need for improved HIV prevention efforts and enhanced care for people living with HIV to improve their long-term health outcomes. Necessary interventions include expanded HIV testing, access and adherence support for PrEP and ART, and measures to reduce TB and noncommunicable disease burden in people with HIV [2, 22, 23]. The prevalence of smokers in this study was lower compared to other studies in South Africa [13, 24, 25]. Smoking prevalence in previous studies in South Africa varies by province, with much higher prevalence in the Western Cape province [24]. Stanton and colleagues examined the relationship between tobacco use and quality of life among adults with depression in Western Cape who were receiving antiretroviral therapy for HIV and found that about 24% of the sample reported current (i.e., daily or weekly) tobacco use, with higher prevalence of current use among men (48.1%) than women (15.5%) [24]. The percentage of current smoking in the Vukuzazi study were high relative to one study that assessed smoking among PLWH in Johannesburg [25] and slightly low relative to another study among PLWH in Klerksdorp [13]. Elf and colleagues found that 52% of men living with HIV in the Klerksdorp sample were defined as currently smoking [25]. A possible difference in the smoking prevalence found in our study versus that in other studies may be due to our predominantly rural population, whereas the other studies were conducted in predominantly urban populations. Differences in sociodemographic factors and regions may account for these discrepancies, as prevalence of cigarette smoking differs greatly by province, with the highest prevalence among men reported in the Northern Cape and the highest prevalence among women reported in the Western Cape [26]. We previously reported that 34.2% had HIV, 1.4% had active tuberculosis, 8.5% had elevated blood glucose, and 23.0% had elevated blood pressure [2].

Overall, our results did not find any association between current smoking and the four diseases among females, due in part to small numbers of female smokers which limits the ability to detect significant associations among females. However, our sex-stratified unadjusted analysis demonstrated significantly higher prevalence of HIV (overall) and uncontrolled (viremic) HIV among men who were currently smoking at the time of the survey compared to men who had never smoked. This could be behavioral, since people who smoke may be less likely to adhere to medication, or biological in that smoking may increase inflammation and thus could conceivably drive viral replication. The methods of the current study cannot distinguish these possibilities, which warrant future investigation. The fact that this relationship did not persist after multiple logistic regression analysis suggests that differences in age and socio-economic status may explain this effect. In keeping with literature, we found that older age was positively associated with diagnosis of hypertension, diabetes, HIV, and active TB. In addition, higher SES (richer) was associated with hypertension and diabetes but not HIV, viral load or active TB among males. However, SES was associated with lower likelihood of being HIV positive and lower likelihood of being virologically controlled. Nonetheless, our results suggest that people who are currently smoking should receive extra attention for services to diagnose and treat HIV. In addition, all PLWH who smoke should receive additional attention to ensure adherence to ART.

In keeping with several studies, we found an association between smoking and active TB [5, 27, 28]. Overall, the prevalence of active pulmonary TB found in this study was in line with results from a national pulmonary TB prevalence survey in South Africa [29], however we found a strikingly high prevalence of active TB among people who currently smoke – 3% – and people with a history of former smoking—6%. Multiple logistic regression analysis demonstrated that in males the relationship between current smoking and active TB did not remain significant when adjusted for age and socio-economic status, and BMI. The fact that this relationship did not persist after multiple logistic regression analysis suggests that differences in age, socio-economic status, and body weight may explain this effect. Nonetheless, from a public health perspective these results indicate that people currently smoking or with a history of smoking should receive TB screening in addition to tobacco cessation interventions.

Our unadjusted data showed lower prevalence of hypertension and diabetes mellitus among men reporting current smoking compared with those who had never smoked. This relationship did not persist after multiple logistic regression adjustments for age, socio-economic status, and BMI. These findings suggest that the lower rates of hypertension and diabetes in current smokers are likely due to differences in age and body size between current, former and never smokers. Our findings contrast with findings from other settings that have associated smoking with hypertension and diabetes [30]. However, our results may be explained by different demographics, duration of smoking over the lifespan, lack of access to health care in our setting and poor health seeking behaviors in males in our setting as found in Uganda [31]. Point estimates of strengths of association with disease were in some cases higher in former smokers than in current smokers. The reasons for this were likely multifactorial and could be due to small numbers of former smokers which resulted in wide confidence intervals and also the older age of the former smoking group. Smoking is a known risk factor for hypertension, diabetes mellitus, and cardiovascular disease; the current study findings should not be interpreted as evidence to encourage smoking as an intervention to reduce the risk of hypertension or diabetes mellitus [32–34].

The current study has several limitations. Firstly, the cross‐sectional design prevents the drawing of causal inferences. Secondly, data were collected from a rural geographic area in a single province, limiting the generalizability of the findings to urban communities and other groups in South Africa. Thirdly, unmeasured confounders, such as family history of hypertension or diabetes mellitus, dietary factors, alcohol use, and physical activity, or residual confounding by age, may have also influenced the relationship. There also might be a survivor bias, such that some who smoked with active hypertension or diabetes mellitus might have died. In addition, in the logistic regression models, there were only a small number of participants who reported former smoking, potentially masking other associations between smoking with CDs and NCDs that may be apparent with larger study sizes. Moreover, it is feasible that healthier individuals were more likely to move out of the surveillance area in recent times, thereby potentially biasing the prevalence of the health outcomes. Furthermore, our findings could partly be attributed to relatively low prevalence of smoking and few outcomes in a study population of relatively young age. In addition, a limitation of the study design is the use of prevalent outcomes. The temporal relationship between smoking and diseases cannot be determined from these findings. Because we categorized smoking status as binary and did not investigate intensity of smoking (e.g., pack-years or cigarettes per day), we could not determine a dose–response effect. While tobacco use has been reported to be a risk factor for TB and cardiovascular disease, there are fewer studies of the intersection of tobacco, TB, HIV, and non-communicable diseases in sub-Saharan Africa; thus, our investigation of a rural population in South Africa offers a different perspective than many prior studies.

## Conclusions

In this population-based sample of rural residents of South Africa, we found higher prevalence of active TB and virologically uncontrolled HIV and lower prevalence of hypertension and diabetes mellitus in those reporting current smoking compared with those reporting former or never smoking. Regression analyses showed an association between smoking with active TB, hypertension and diabetes in males but not in females. These associations require further exploration, including longitudinal studies, and targeted health screening and tobacco cessation interventions in South Africa to reduce the complications of HIV and TB. The higher prevalence (3–6%) of active TB among those reporting current or former smoking is particularly noteworthy and suggests that these populations be screened more frequently for TB in TB-endemic regions. Combining tobacco cessation interventions with TB screening and/or antiretroviral adherence interventions may provide important individual and public health benefits.

## Supplementary Information

Supplementary Material 1.

## Data Availability

Data available in a publicly accessible repository (https://data.ahri.org/index.php/catalog/1006) which is managed in terms of AHRI’s Data Access Policy (https://www.ahri.org/).
