# Supplementary material for: Association between tobacco smoking and prevalence of HIV, tuberculosis, hypertension and diabetes in rural South Africa: a cross-sectional study
Source: BMC Public Health. 2024 Nov 27;24:3306. doi: 10.1186/s12889-024-20791-4 (PMC11603742; doi:10.1186/s12889-024-20791-4)
Supplement: Supplementary file 1 — Supplementary Material 1. [file 12889_2024_20791_MOESM1_ESM.docx]

**Supplementary Table 1: Communicable and non-communicable diseases by smoking category for females**

| \| **VARIABLE** \| **Total** \| **Current smoking** \| **Former smoking** \| **Never smoking** \|  \| \| --- \| --- \| --- \| --- \| --- \| --- \| \|  \| (N = 12,224) \| (N = 124) \| (N = 49) \| (N = 12,051) \| **P-value** \| |
| --- | --- | --- | --- | --- | --- | --- | --- | --- | --- | --- | --- | --- |
| \| **HIV ELISA Result** \|  \|  \|  \|  \| 0.132 \| \| --- \| --- \| --- \| --- \| --- \| --- \| \| Positive \| 4,690 (38.5%) \| 57 (46.0%) \| 22 (45.8%) \| 4,611 (38.4%) \|  \| \| Negative \| 7,488 (61.5%) \| 67 (54.0%) \| 26 (54.2%) \| 7,395 (61.6%) \|  \| \| **Viral load category** \|  \|  \|  \|  \| 0.570 \| \| <400 copies/mL \| 4,019 (85.2%) \| 46 (80.7%) \| 18 (81.8%) \| 3,955 (85.3%) \|  \| \| >=400 copies/mL \| 699 (14.8%) \| 11 (19.3%) \| 4 (18.2%) \| 684 (14.7%) \|  \| \| **Active tuberculosis** \|  \|  \|  \|  \| 0.248 \| \| Yes \| 134 (1.2%) \| 3 (2.6%) \| 1 (2.6%) \| 130 (1.2%) \|  \| \| No \| 11,303 (98.8%) \| 112 (97.4%) \| 37 (97.4%) \| 11,152 (98.8%) \|  \| \| **Hypertension** \|  \|  \|  \|  \| 0.734 \| \| Yes \| 3,561 (29.2%) \| 33 (26.8%) \| 16 (32.7%) \| 3,512 (29.2%) \|  \| \| No \| 8,630 (70.8%) \| 90 (73.2%) \| 33 (67.3%) \| 8,507 (70.8%) \|  \| \| **Diabetes mellitus** \|  \|  \|  \|  \| 0.060 \| \| Yes \| 1,422 (11.7%) \| 8 (6.5%) \| 9 (18.8%) \| 1,405 (11.7%) \|  \| \| No \| 10,761 (88.3%) \| 116 (93.5%) \| 39 (81.3%) \| 10,606 (88.3%) \|  \| |

**Excluding former smokers (previous main analysis)**

**Supplementary Table 2: Association between communicable and non-communicable diseases and smoking status, multiple logistic regression.**

| **VARIABLE** | **MALES** | | **FEMALES** | |
| --- | --- | --- | --- | --- |
|  | **Adjusted odds ratio (95% CI)** | **P-value** | **Adjusted odds ratio (95% CI)** | **P-value** |
| **HIV (positive ELISA result)** |  |  |  |  |
| Never smoking | Reference |  | Reference |  |
| Current smoking | 1.10 (0.93; 1.30) | 0.272 | 1.25 (0.83; 1.89) | 0.278 |
| **Viral load <400 copies/mL among those with HIV** |  |  |  |  |
| Never smoking | Reference |  | Reference |  |
| Current smoking | 0.85 (0.63; 1.15) | 0.291 | 0.87 (0.43; 1.77) | 0.698 |
| **Active tuberculosis** |  |  |  |  |
| Never smoking | Reference |  | Reference |  |
| Current smoking | 1.95 (1.22; 3.13) | 0.006 | 1.95 (0.58; 6.53) | 0.280 |
| **Hypertension** |  |  |  |  |
| Never smoking | Reference |  | Reference |  |
| Current smoking | 0.67 (0.54; 0.83) | <0.001 | 0.80 (0.49; 1.30) | 0.366 |
| **Diabetes mellitus** |  |  |  |  |
| Never smoking | Reference |  | Reference |  |
| Current smoking | 0.38 (0.24; 0.61) | <0.001 | 0.63 (0.30; 1.34) | 0.227 |

For each outcome variable (HIV, viral load <400 copies/mL, tuberculosis, hypertension, and diabetes mellitus), we ran separate multiple logistic regression models, adjusting for age and household socio-economic status. All logistic regression models excluded former smoking due to small numbers in the category. CI: Confidence Interval

**Supplementary Table 3: Association between communicable and non-communicable diseases and smoking status, multiple logistic regression, including BMI.**

| **VARIABLE** | **MALES** | | **FEMALES** | |
| --- | --- | --- | --- | --- |
|  | **Adjusted odds ratio (95% CI)** | **P-value** | **Adjusted odds ratio (95% CI)** | **P-value** |
| **HIV (positive ELISA result)** |  |  |  |  |
| Never smoking | Reference |  | Reference |  |
| Current smoking | 0.98 (0.83; 1.17) | 0.860 | 1.08 (0.71; 1.64) | 0.725 |
| **Viral load <400 copies/mL among those with HIV** |  |  |  |  |
| Never smoking | Reference |  | Reference |  |
| Current smoking | 0.89 (0.65; 1.21) | 0.454 | 0.88 (0.43; 1.79) | 0.719 |
| **Active tuberculosis** |  |  |  |  |
| Never smoking | Reference |  | Reference |  |
| Current smoking | 1.44 (0.89; 2.34) | 0.136 | 1.46 (0.42; 5.10) | 0.544 |
| **Hypertension** |  |  |  |  |
| Never smoking | Reference |  | Reference |  |
| Current smoking | 0.77 (0.61; 0.96) | 0.020 | 0.95 (0.58; 1.57) | 0.967 |
| **Diabetes mellitus** |  |  |  |  |
| Never smoking | Reference |  | Reference |  |
| Current smoking | 0.51 (0.31; 0.83) | 0.006 | 0.87 (0.40; 1.88) | 0.718 |

For each outcome variable (HIV, viral load <400 copies/mL, tuberculosis, hypertension, and diabetes mellitus), we ran separate multivariable logistic regression models, adjusting for age, body mass index, and household socio-economic status. All logistic regression models excluded former smoking due to small numbers in the category. CI: Confidence Interval.

**Including former smokers with never smokers**

**Supplementary Table 4: Association between communicable and non-communicable diseases and smoking status, multiple logistic regression (former smokers included with never smokers).**

| **VARIABLE** | **MALES** | | **FEMALES** | |
| --- | --- | --- | --- | --- |
|  | **Adjusted odds ratio (95% CI)** | **P-value** | **Adjusted odds ratio (95% CI)** | **P-value** |
| **HIV (positive ELISA result)** |  |  |  |  |
| Non-current smoking | Reference |  | Reference |  |
| Current smoking | 1.10 (0.93; 1.30) | 0.262 | 1.25 (0.83; 1.88) | 0.289 |
| **Viral load <400 copies/mL among those with HIV** |  |  |  |  |
| Non-current smoking | Reference |  | Reference |  |
| Current smoking | 0.83 (0.62; 1.13) | 0.234 | 0.87 (0.43; 1.77) | 0.701 |
| **Active tuberculosis** |  |  |  |  |
| Non-current smoking | Reference |  | Reference |  |
| Current smoking | 1.90 (1.20; 3.03) | 0.007 | 1.98 (0.59; 6.61) | 0.269 |
| **Hypertension** |  |  |  |  |
| Non-current smoking | Reference |  | Reference |  |
| Current smoking | 0.68 (0.55; 0.85) | <0.001 | 0.80 (0.49; 1.31) | 0.374 |
| **Diabetes mellitus** |  |  |  |  |
| Non-current smoking | Reference |  | Reference |  |
| Current smoking | 0.39 (0.25; 0.62) | <0.001 | 0.63 (0.30; 1.33) | 0.220 |

For each outcome variable (HIV, viral load <400 copies/mL, tuberculosis, hypertension, and diabetes mellitus), we ran separate multiple logistic regression models, adjusting for age and household socio-economic status. CI: Confidence Interval

**Supplementary Table 5: Association between communicable and non-communicable diseases and smoking status, multiple logistic regression, including BMI (former smokers included with never smokers).**

| **VARIABLE** | **MALES** | | **FEMALES** | |
| --- | --- | --- | --- | --- |
|  | **Adjusted odds ratio (95% CI)** | **P-value** | **Adjusted odds ratio (95% CI)** | **P-value** |
| **HIV (positive ELISA result)** |  |  |  |  |
| Non-current smoking | Reference |  | Reference |  |
| Current smoking | 0.98 (0.82; 1.16) | 0.819 | 1.07 (0.71; 1.63) | 0.746 |
| **Viral load <400 copies/mL among those with HIV** |  |  |  |  |
| Non-current smoking | Reference |  | Reference |  |
| Current smoking | 0.87 (0.64; 1.18) | 0.375 | 0.88 (0.43; 1.80) | 0.731 |
| **Active tuberculosis** |  |  |  |  |
| Non-current smoking | Reference |  | Reference |  |
| Current smoking | 1.59 (0.99; 2.56) | 0.055 | 1.61 (0.48; 5.38) | 0.443 |
| **Hypertension** |  |  |  |  |
| Non-current smoking | Reference |  | Reference |  |
| Current smoking | 0.78 (0.63; 0.98) | 0.030 | 0.95 (0.58; 1.57) | 0.856 |
| **Diabetes mellitus** |  |  |  |  |
| Non-current smoking | Reference |  | Reference |  |
| Current smoking | 0.52 (0.32; 0.84) | 0.008 | 0.86 (0.40; 1.85) | 0.698 |

For each outcome variable (HIV, viral load <400 copies/mL, tuberculosis, hypertension, and diabetes mellitus), we ran separate multivariable logistic regression models, adjusting for age, body mass index, and household socio-economic status. CI: Confidence Interval.
